# Molecular mechanisms of emerging inflammasome complexes and their activation and signaling in inflammation and pyroptosis

**DOI:** 10.1111/imr.13406

**Published:** 2024-10-01

**Authors:** Abhimanu Pandey, Zheyi Li, Manjul Gautam, Aritra Ghosh, Si Ming Man

**Affiliations:** ^1^ Division of Immunology and Infectious Diseases, The John Curtin School of Medical Research The Australian National University Canberra Australia

**Keywords:** autoimmunity, autoinflammation, bacteria, cancer, caspase‐1, caspase‐11, caspase‐4, caspase‐5, cell death, cytokines, gasdermin D, immunity, infection, inflammatory caspases, interferons, lipopolysaccharide, PANoptosis, PANoptosome, parasites, pattern‐recognition receptors, viruses

## Abstract

Inflammasomes are multi‐protein complexes that assemble within the cytoplasm of mammalian cells in response to pathogen‐associated molecular patterns (PAMPs) or damage‐associated molecular patterns (DAMPs), driving the secretion of the pro‐inflammatory cytokines IL‐1β and IL‐18, and pyroptosis. The best‐characterized inflammasome complexes are the NLRP3, NAIP‐NLRC4, NLRP1, AIM2, and Pyrin canonical caspase‐1‐containing inflammasomes, and the caspase‐11 non‐canonical inflammasome. Newer inflammasome sensor proteins have been identified, including NLRP6, NLRP7, NLRP9, NLRP10, NLRP11, NLRP12, CARD8, and MxA. These inflammasome sensors can sense PAMPs from bacteria, viruses and protozoa, or DAMPs in the form of mitochondrial damage, ROS, stress and heme. The mechanisms of action, physiological relevance, consequences in human diseases, and avenues for therapeutic intervention for these novel inflammasomes are beginning to be realized. Here, we discuss these emerging inflammasome complexes and their putative activation mechanisms, molecular and signaling pathways, and physiological roles in health and disease.

## 1 INTRODUCTION

Inflammation is a fundamental host defense mechanism triggered in response to pathogens and tissue injury. This process relies on cells continuously surveilling the body for signs of infection and/or damage. Pattern‐recognition receptors (PRRs) play a central role in detecting molecular structures found on microorganisms called pathogen‐associated molecular patterns (PAMPs), or endogenous molecules released from damaged or dying cells called damage‐associated molecular pattern (DAMPs).^1^, ^2^ PRRs then trigger the activation of inflammatory signaling pathways that signify the beginning of an inflammatory response.^2^

**FIGURE 1:**
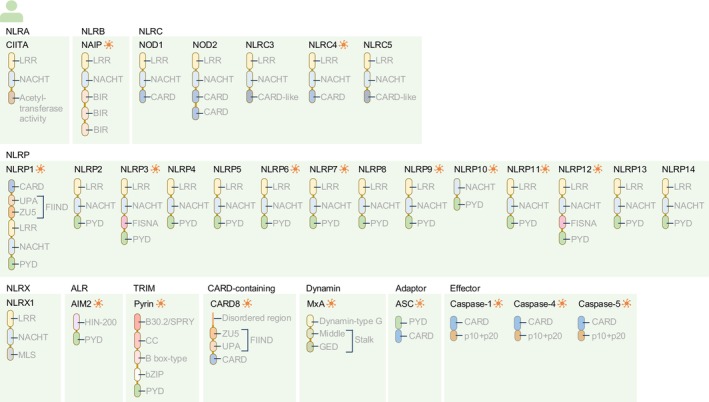
Inflammasome‐forming proteins and related molecules in humans. Proteins that have been demonstrated to form an inflammasome are indicated by an orange‐colored wheel‐like symbol. The nucleotide‐binding domain and leucine‐rich repeat (LRR) containing gene family (NLR) are grouped based on their N‐terminal domains. At least 22 NLRs have been identified in humans. NLRA is defined by an acetyl‐transferase activity or acidic transactivation domain and has the sole member Class II, major histocompatibility complex, transactivator (CIITA). NLRB is defined by a baculoviral inhibitory repeat (BIR)‐like domain. The NLRB subfamily has one member called NLR family member apoptosis inhibitory protein (NAIP). NLRC is defined by a caspase recruitment domain (CARD) or a CARD‐like disordered region. This family contains five members: Nucleotide‐binding oligomerization domain containing 1 (NOD1, also known as NLRC1), nucleotide‐binding oligomerization domain containing 2 (NOD2, also known as NLRC2), NLRC3, NLRC4, and NLRC5. NLRP is defined by a pyrin domain (PYD). This family contains 14 members in humans: NLRP1, NLRP2, NLRP3, NLRP4, NLRP5, NLRP6, NLRP7, NLRP8, NLRP9, NLRP10, NLRP11, NLRP12, NLRP13, and NLRP14. NLRX is an NLR family with the sole member NLRX1 that carries an N‐terminal domain with no strong homology to the N‐terminal domain of any other NLR subfamily member. The N‐terminal region carries a mitochondrial localization signal (MLS). The AIM2‐like receptor (ALR) family contains the founding member called absent in melanoma 2 (AIM2). The tripartite motif‐containing (TRIM) family is defined by an N‐terminal set of domains known as the RING, B‐box‐type, coiled‐coil (CC) motif. This family has one inflammasome‐forming protein called Pyrin (also known as Mediterranean fever, MEFV or TRIM20). A CARD‐containing protein called CARD8 can form an inflammasome. The Dynamin family of large GTPases contains an inflammasome‐forming protein called Myxovirus resistance protein 1 (MxA, also known as MX1). Inflammasome assembly requires the adaptor protein called apoptosis‐associated speck‐like protein containing a CARD (ASC, also known as PYCARD). The proteases that induce proteolytic cleavage of inflammasome substrates in humans are caspase‐1, caspase‐4 and caspase‐5. bZIP, Basic Leucine Zipper domain; FIIND, Function‐to‐Find Domain; FISNA, Fish‐specific NACHT‐associated domain; GED, GTPase effector domain; HIN‐200, Hematopoietic IFN‐inducible Nuclear proteins with a 200‐amino acid motif; NACHT, Domain present in NAIP, CIITA, HET‐E, and TP‐1; SPRY, SPla/Ryanodine receptor domain; UPA, UNC5, PIDD, and Ankirins domain; ZU5, ZO‐1, and UNC5 domain. The domain structures are annotated based on UniProt, NCBI, and the published literature.

**FIGURE 2:**
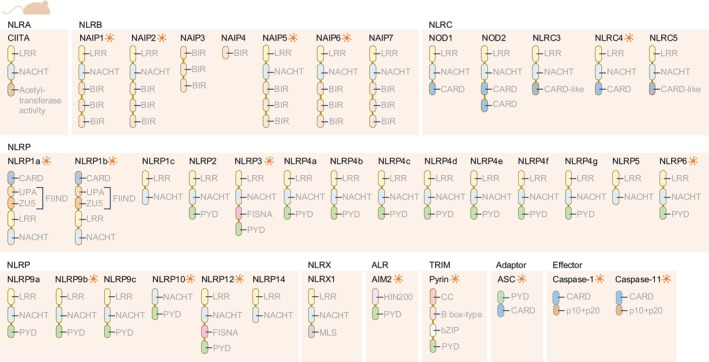
Inflammasome‐forming proteins and related molecules in mice. Proteins that have been demonstrated to form an inflammasome are indicated by an orange‐colored wheel‐like symbol. The nucleotide‐binding domain and leucine‐rich repeat (LRR) containing gene family (NLR) are grouped based on their N‐terminal domains. At least 34 NLRs have been identified in mice. NLRA is defined by an acetyl‐transferase activity or acidic transactivation domain and has a single‐member Class II, major histocompatibility complex, transactivator (CIITA). NLRB is defined by a baculoviral inhibitory repeat (BIR)‐like domain. This subfamily has seven members of NLR family member apoptosis inhibitory protein (NAIP): NAIP1, NAIP2, NAIP3, NAIP4, NAIP5, NAIP6, and NAIP7. NLRC is defined by a caspase recruitment domain (CARD) or a CARD‐like disordered region. This family contains five members: Nucleotide‐binding oligomerization domain containing 1 (NOD1, also known as NLRC1), nucleotide‐binding oligomerization domain containing 2 (NOD2, also known as NLRC2), NLRC3, NLRC4, and NLRC5. NLRP is defined by a pyrin domain (PYD), however, of the 20 members in mice, four do not carry a PYD. This family contains NLRP1a, NLRP1b, NLRP1c, NLRP2, NLRP3, NLRP4a, NLRP4b, NLRP4c, NLRP4d, NLRP4e, NLRP4f, NLRP4g, NLRP5, NLRP6, NLRP9a, NLRP9b, NLRP9c, NLRP10, NLRP12, and NLRP14. NLRX is an NLR family with the sole member NLRX1 that carries an N‐terminal domain with no strong homology to the N‐terminal domain of any other NLR subfamily member. The N‐terminal region carries a mitochondrial localization signal (MLS). The AIM2‐like receptor (ALR) family contains the founding member called absent in melanoma 2 (AIM2). The tripartite motif‐containing (TRIM) family is defined by an N‐terminal set of domains known as the RING, B‐box‐type, coiled‐coil (CC) motif. This family has one inflammasome‐forming protein called Pyrin (also known as Mediterranean fever, MEFV or TRIM20). Inflammasome assembly requires the adaptor protein called apoptosis‐associated speck‐like protein containing a CARD (ASC, also known as PYCARD). The proteases that induce proteolytic cleavage of inflammasome substrates in mice are caspase‐1 and caspase‐11. bZIP, Basic Leucine Zipper domain; FIIND, Function‐to‐Find Domain; FISNA, Fish‐specific NACHT‐associated domain; HIN‐200, Hematopoietic IFN‐inducible Nuclear proteins with a 200‐amino acid motif; NACHT, Domain present in NAIP, CIITA, HET‐E, and TP‐1; UPA, UNC5, PIDD, and Ankirins domain; ZU5, ZO‐1, and UNC5 domain. The domain structures are annotated based on UniProt, NCBI and the published literature.

**FIGURE 3:**
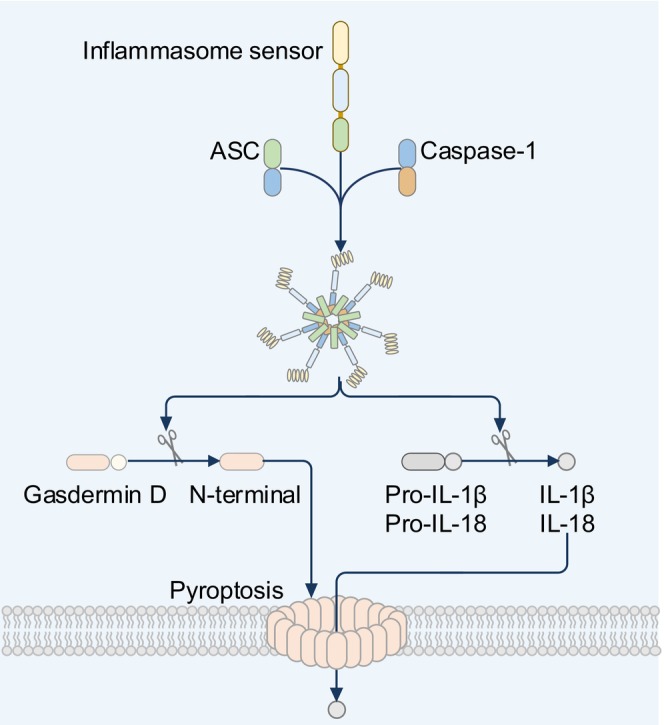
A high‐level overview of inflammasome assembly and activation in mammalian cells. Inflammasome sensors can interact directly with their target ligand or respond to a variety of physiological changes. Following activation, most inflammasome sensors recruit an adaptor protein called apoptosis‐associated speck‐like protein containing a caspase‐recruitment domain (also known as ASC or PYCARD) and the cysteine protease caspase‐1. Activated caspase‐1 cleaves gasdermin D to release the N‐terminal domain which induces pyroptosis. Caspase‐1 also cleaves pro‐interleukin (IL)‐1β and pro‐IL‐18 into their biologically active forms which are released through gasdermin D pores.

An important class of PRRs is the cytoplasmic inflammasome sensors, which include members of the nucleotide‐binding domain (NBD) and leucine‐rich repeat (LRR) containing gene family (NLR), AIM2‐like receptor family (ALR) and Pyrin (Figures 1 and 2). The NLR family has the largest number of inflammasome sensors. Of the 22 NLR proteins in humans, 10 of them have been shown to form inflammasomes (Figure 1). Of the 34 NLR proteins in mice, 12 of them can assemble inflammasomes (Figure 2). The inflammasome protein scaffold comprises three core components: one or more inflammasome sensor proteins, the adaptor protein apoptosis‐associated speck‐like protein containing a caspase‐recruitment domain (also known as ASC or PYCARD), and an enzymatic effector protein caspase‐1 (Figure 3).^3^, ^4^ Caspase‐1 from the inflammasome drives the proteolytic cleavage of the proinflammatory cytokines pro‐IL‐1β and pro‐IL‐18, resulting in the release of bioactive IL‐1β and IL‐18. Caspase‐1 also cleaves the pore‐forming protein gasdermin D, liberating its N‐terminal fragment that oligomerizes to form pores on the plasma membrane, through which bioactive IL‐1β and IL‐18 can escape from within the cell to the extracellular environment (Figure 3).^5^, ^6^ In addition to the canonical inflammasome scaffold structure, a non‐canonical inflammasome pathway has been discovered.^7^ This pathway is defined by the activation of mouse caspase‐11 or human caspase‐4 and caspase‐5 following sensing of cytoplasmic lipopolysaccharide (LPS).^8^ These caspases directly induce the proteolytic cleavage of gasdermin D.^5^, ^6^, ^9^ Canonical and non‐canonical inflammasome activation both lead to the formation of gasdermin D pores and the inflammatory lytic cell death modality called pyroptosis. Pyroptotic cells liberate PAMPs and DAMPs that activate PRRs on neighboring cells to further perpetuate inflammation, inflammasome activation, and PANoptosome activation.^10^ These biological events represent evolutionarily conserved mechanisms to drive a rapid inflammatory response, cell death, pathogen clearance, and the initiation of adaptive immune responses.^11^

Over the past two decades, important strides have been made in understanding the roles and mechanisms of inflammasomes in maintaining homeostasis and the development and progression of inflammatory diseases. The best characterized inflammasome complexes are the NLRP3, NAIP‐NLRC4, NLRP1, AIM2, and Pyrin inflammasomes, and the caspase‐11 non‐canonical inflammasome. These inflammasome complexes have been reviewed extensively across multiple disease contexts,^12^, ^13^, ^14^, ^15^, ^16^, ^17^, ^18^, ^19^, ^20^, ^21^, ^22^, ^23^, ^24^, ^25^, ^26^, ^27^, ^28^, ^29^, ^30^, ^31^ and will not be discussed in this review. Newer inflammasome sensor proteins have been identified, including NLRP6, NLRP7, NLRP9, NLRP10, NLRP11, NLRP12, CARD8, and MxA. These inflammasome sensors have remained under‐characterized with respect to their mechanisms of action, physiological and disease relevance, and their ability to be targeted for therapeutic intervention. Here, we provide an overview of these emerging and newer inflammasome complexes and their putative activators, molecular mechanisms of activation, and physiological roles in health and disease.

## 2 NLRP6

**FIGURE 4:**
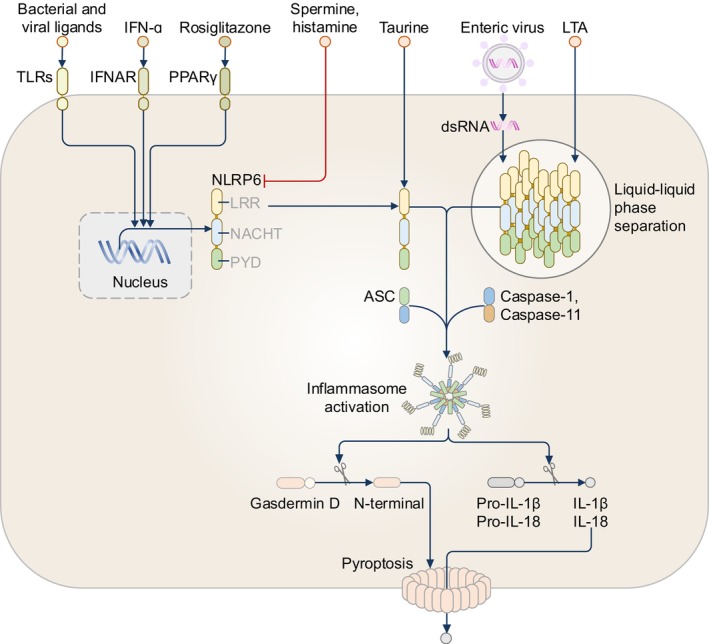
The NLRP6 inflammasome. The expression of NLRP6 is induced by microbial ligands, type I interferons (IFN‐α) and peroxisome proliferator‐activated receptor‐γ (PPARγ) agonist, rosiglitazone. These ligands activate mammalian cells via their respective cell‐surface receptors: Toll‐like receptors (TLRs), Interferon alpha/beta receptor (IFNAR), or PPARγ. The metabolites histamine and spermine attenuate the activation of the NLRP6 inflammasome, whereas the metabolite taurine promotes the activation of the NLRP6 inflammasome. NLRP6 can undergo liquid–liquid phase separation upon interaction with double‐stranded (ds)RNA from enteric viruses or lipoteichoic acid (LTA) from Gram‐positive bacteria leading to inflammasome activation. In the context of the activation of NLRP6 by LTA, NLRP6 drives the recruitment of caspase‐1 and caspase‐11 to the same inflammasome complex.

NLRP6, also known as NALP6 or PYPAF5, is predominantly expressed in the human and mouse intestine, and to a lesser extent in the liver, brain, kidney, and lungs.^32^ The cellular turnover of NLRP6 is, in part, controlled at the transcriptional level, indicating that the activation of NLRP6 may be sensitized by a priming event similar to that in NLRP3 (Figure 4). In this context, the peroxisome proliferator‐activated receptor‐γ agonist, rosiglitazone, has been shown to induce the expression of NLRP6 in the mouse small intestine.^33^, ^34^ The ssRNA virus encephalomyocarditis virus, type I interferons, and synthetic dsRNA polyI:C also induce the transcription of the gene encoding NLRP6 in mouse embryonic fibroblasts.^35^

An earlier study found that co‐expression of human NLRP6 and ASC in HEK293T or monkey kidney fibroblast‐like cell line COS‐7L activates caspase‐1 and induces the secretion of IL‐1β,^36^ hinting that NLRP6 might assemble an inflammasome. Further studies in mouse models of colitis and colorectal cancer demonstrated that NLRP6 functions as an inflammasome and drives IL‐18 release to strengthen epithelial barrier integrity.^37^, ^38^, ^39^ In mouse intestinal epithelial cells, NLRP6 senses the microbiota‐derived organic acid, taurine, and forms an inflammasome, promoting the release of IL‐18 and anti‐microbial molecules ITLN1, RELMβ, and angiogenin‐4.^40^ Moreover, NLRP6‐dependent IL‐18 has been shown to restrict the intestinal parasite *Cryptosporidium tyzzeri* in mouse enterocytes.^41^ In mouse goblet cells, NLRP6 drives the release of IL‐18 and promotes mucus secretion in response to Toll‐like receptor (TLR) ligands and prevents the invasion of the pathogenic bacterium *Citrobacter rodentium*.^42^, ^43^ However, another study revealed that mucus layer formation is not dependent on the NLRP6 inflammasome and IL‐18.^44^ While taurine triggers NLRP6 inflammasome assembly, metabolites such as histamine and spermine suppress NLRP6 activation in mice,^40^ highlighting that microbiota‐derived metabolites shape intestinal homeostasis by activating and inhibiting the NLRP6 inflammasome (Figure 4). Further, the deubiquitinase CYLD was found to prevent excessive production of NLRP6‐dependent IL‐18 in mouse colonic epithelial cells.^45^ CYLD binds to NLRP6 and induces post‐translational deubiquitination of NLRP6 by cleaving the K63‐linked ubiquitin chain on NLRP6,^45^ signifying a host‐level control of NLRP6 inflammasome to prevent aberrant activation.

The relationship between NLRP6 and the microbiome is not always straightforward. In non‐littermate‐controlled mice, NLRP6 expression appears to affect the composition of the gut microbiome.^37^ Other studies analyzing littermate‐controlled wild‐type (WT) and mice lacking NLRP6 have shown that NLRP6 does not affect the profile of the gut microbiome.^46^, ^47^ These studies underscore the necessity to integrate littermate breeding strategies to analyze the gene effects on intestinal health and disease.^48^, ^49^

Under what conditions NLRP6 assembles the inflammasome during microbial infections has still not been firmly established. In response to infection with *Listeria monocytogenes*, *Salmonella enterica* serovar Typhimurium (*S*. Typhimurium) or *Escherichia coli*, WT mice and mice lacking NLRP6 have similar levels of caspase‐1 activation and IL‐1β release.^50^ However, in mice undergoing cecal ligation and puncture‐induced polymicrobial sepsis or *E*. *coli*‐induced sepsis, NLRP6‐mediated release of IL‐18 exacerbates sepsis.^51^ Other studies have shown a reduction in caspase‐1 activation and IL‐1β secretion in bone‐marrow‐derived macrophage (BMDMs) lacking NLRP6 compared to WT BMDMs following infection with *Staphylococcus aureus* or *L*. *monocytogenes*.^52^, ^53^ Further examples that NLRP6 can assemble an inflammasome are reported in mouse dendritic cells infected with the parasite *Schistosoma mansoni*, mouse lung cells infected with the bacterium *Klebsiella pneumoniae*, and mouse peritoneal macrophages and neutrophils infected with the bovine pathogen *Pasteurella multocida*.^54^, ^55^, ^56^

Besides taurine, other microbial ligands triggering the assembly of the NLRP6 inflammasome have been proposed (Figure 4). Stimulation of mouse BMDMs with a major constituent of the Gram‐positive bacterial cell wall, lipoteichoic acid (LTA), leads to NLRP6‐dependent activation of caspase‐11, leading to the activation of caspase‐1 and release of IL‐1β and IL‐18.^53^ In this context, NLRP6 causes the dual recruitment of caspase‐1 and caspase‐11 to the same inflammasome complex,^53^ but the role of caspase‐11 in this complex is unclear. Caspase‐11 might contribute to a scaffolding role within the protein complex rather than sensing LTA, because the glycerophosphate repeats of the LTA was found to directly bind to the LRR of NLRP6, leading to the recruitment and oligomerization of ASC (Figure 4).^53^ Another study argued that NLRP6 is not expressed in BMDMs, but confirmed that co‐expression of NLRP6, ASC and caspase‐1 in HEK293T/17 cells results in the proteolytic cleavage of IL‐1β.^57^ Co‐expression of caspase‐11 in these cells does not further potentiate the proteolytic cleavage of IL‐1β,^57^ implying either no requirement for caspase‐11 in NLRP6 inflammasome activation or the role of caspase‐11 in the NLRP6 inflammasome is cell‐type‐specific.

LPS has also been proposed as a potential ligand of NLRP6. Human NLRP6 co‐localizes with ASC in HeLa cells transfected with *E*. *coli* LPS, with the LRR of NLRP6 proposed to interact with LPS.^58^ A later study revealed using an in vitro ligand binding assay that NLRP6 binds to dsRNA and LTA, but not LPS.^59^ In mouse embryonic fibroblast cell lines and human lymphoma cell line MBL‐1 stably expressing NLRP6 and treated with the synthetic dsRNA polyI:C, NLRP6 undergoes liquid–liquid phase separation and assembles a dsRNA‐NLRP6‐ASC speck leading to caspase‐1 activation.^59^ Mice lacking NLRP6 or harboring a *Nlrp6* ^K350‐354A^ gene that encodes NLRP6 carrying a mutation in the NACHT domain preventing phase separation of NLRP6, called *Nlrp6* ^K350‐354A^ mice, and challenged with the ssRNA virus mouse Hepatitis virus or dsRNA virus Rotavirus have a reduction in the proteolytic cleavage of caspase‐1 and secretion of IL‐18,^59^ supporting the concept that phase separation of NLRP6 promotes inflammasome activation (Figure 4).

Cryogenic electron microscopy analysis of the crystal structure of the pyrin domain (PYD) of human NLRP6 showed that NLRP6 assembles filamentous structures and recruits ASC via PYD–PYD interactions.^60^ Full‐length human NLRP6 is less efficient in nucleating ASC compared to the PYD of NLRP6 and PYD‐NBD of NLRP6, owing to the auto‐inhibitory nature of the LRR on monomeric NLRP6.^60^ These findings suggest the possibility that NLRP6 undergoes a conformational change after ligand binding,^53^ to overcome auto‐inhibition prior to liquid–liquid phase separation.

## 3 NLRP7

**FIGURE 5:**
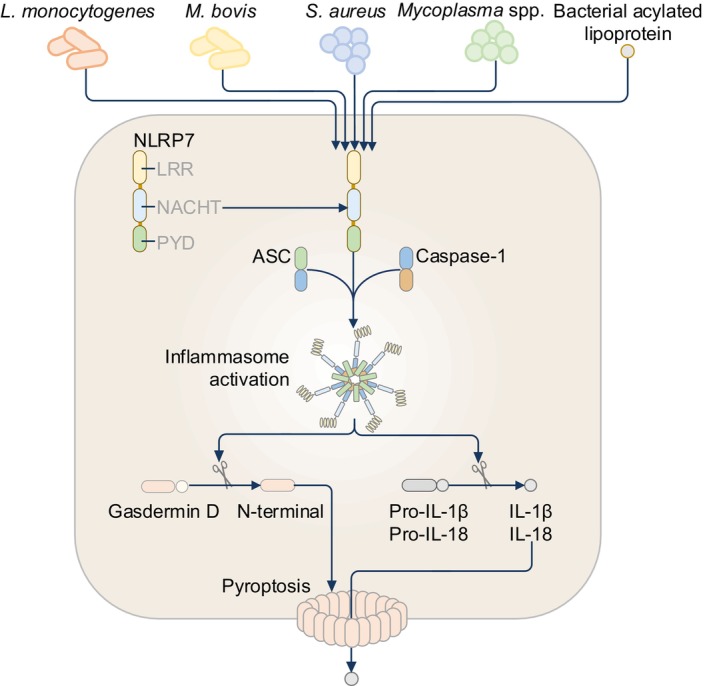
The NLRP7 inflammasome. The NLRP7 inflammasome is activated in human macrophages following infection with *Listeria monocytogenes*, *Mycobacterium bovis*, *Staphylococcus aureus* and *Mycoplasma* species. NLRP7 also senses the bacterial acylated lipoprotein of *Mycoplasma* species, followed by interaction with ASC and the recruitment of caspase‐1 to assemble a functional inflammasome complex.

The gene encoding NLRP7 (also known as NALP7, NOD12, PAN7, PYPAF3, CLR19.4, and HYDM) is located on human chromosome 19q13.42, but mice do not carry a gene for NLRP7.^61^, ^62^, ^63^ NLRP7 is expressed in immune organs such as the spleen, thymus, bone marrow, and the nervous system.^64^ The functions of NLRP7 are still largely a mystery, but an NLRP7 inflammasome complex has been described (Figure 5). In response to bacterial pathogens leading to inflammasome responses in human macrophages,^65^, ^66^, ^67^ NLRP7 senses the bacterial acylated lipoprotein of *Mycoplasma* species, followed by interaction with ASC and the recruitment of caspase‐1 to assemble a functional inflammasome complex for the maturation of IL‐1β and IL‐18.^65^ Moreover, the NLRP7 inflammasome is also activated in macrophages following infection with *S*. *aureus*, *L*. *monocytogenes*, and *Mycobacterium bovis* (Figure 5).^65^, ^67^ Additional insights into the mechanisms of activation identified that the NBD of NLRP7 binds to ATP and acts as an ATPase during infection with *S*. *aureus*.^66^ Furthermore, the intact nucleotide‐binding motif Walker A within the NBD of NLRP7 is essential for nucleotide binding and hydrolysis, and oligomerization of NLRP7.^66^ However, overexpression and chemical‐induced dimerization of the PYD of NLRP7 in the presence of ASC and caspase‐1 in HEK293T/17 cells do not drive the proteolytic cleavage of gasdermin D or pro‐IL‐1β.^57^ Further, co‐expression of NLRP7 and ASC in HEK293T cells does not lead to ASC speck formation,^68^ in contrast to that observed in HEK293 cells.^65^ It is possible that co‐factors required to mediate the formation of the NLRP7 inflammasome are not expressed in HEK293T cells or their subvariant and derivative cell lines. In another overexpression system in HEK293 cells, NLRP7 interacts with pro‐caspase‐1 or pro‐IL‐1β but causes a reduction in IL‐1β release.^64^ Findings from overexpression systems based on HEK293 cells and their derivatives need further validation in more physiologically relevant systems, given that NLRP7 does not inhibit IL‐1β release in other cell types, such as THP‐1 cells and fibroblasts.^65^, ^66^, ^67^, ^69^

Since the first report of a mutation in the gene encoding NLRP7 being linked to the human disease hydatidiform mole in 1999,^70^ more than 200 unique sequence variants of *NLRP7* associated with this condition have now been identified.^71^ Hydatidiform mole is a type of gestational trophoblastic disease characterized by abnormal trophoblast proliferation.^72^, ^73^ Studies have suggested that NLRP7 most likely contributes to the development of trophoblasts and disease progression owing to NLRP7 regulating the bone morphogenic signaling protein 4 pathway.^72^, ^73^, ^74^, ^75^ However, the molecular mechanisms and functional effects of these mutations of NLRP7 in causing hydatidiform mole are not known.

## 4 NLRP9

Humans carry a single gene encoding NLRP9 (also known as NALP9, NOD6, and PAN12), whereas mice have three paralogs encoding NLRP9a, NLRP9b and NLRP9c (Figures 1 and 2).^76^ Human and mouse NLRP9 are mainly expressed in the reproductive system, including oocysts, ovaries, and testes.^77^ The gene encoding human NLRP9 is also expressed in brain endothelial cells, microglial cells, and pericytes, and mouse NLRP9b is expressed in lung cells and intestinal epithelial cells.^77^, ^78^

**FIGURE 6:**
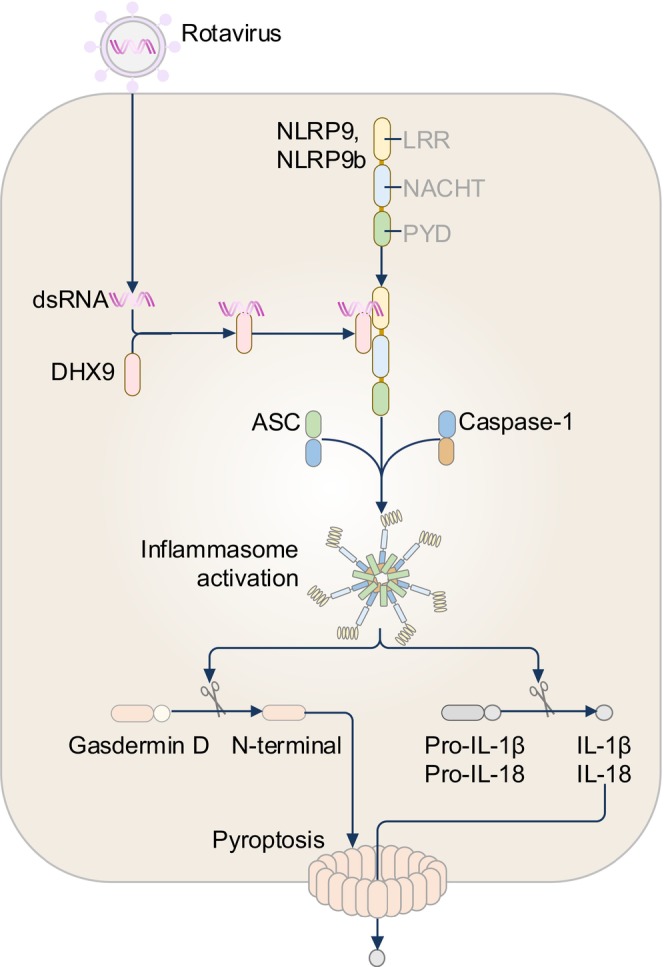
The NLRP9 inflammasome. The mouse NLRP9b inflammasome is activated in intestinal epithelial cells during rotavirus infection. The double‐stranded (ds)RNA of rotavirus is recognized by the RNA sensor DEAH‐box helicase (DHX) 9, which binds to mouse NLRP9b, potentiating the activation of the inflammasome. In addition, human NLRP9 also co‐precipitates with dsRNA, DHX9 and ASC in HEK293T cells.

Upon infection with the dsRNA virus Rotavirus, mice lacking NLRP9b specifically in intestinal epithelial cells have reduced cleavage of caspase‐1 and IL‐18 release, and a higher viral load compared to WT mice,^79^ providing the first evidence in a physiological model that NLRP9b can form an inflammasome complex (Figure 6). NLRP9b does not detect the ligand from Rotavirus directly; instead, the RNA helicase DHX9 binds dsRNA from Rotavirus and associates with NLRP9b to initiate inflammasome assembly (Figure 6).^79^ In HEK293T cells infected with Rotavirus, NLRP9, but not NLRP9b interacts with ASC and caspase‐1.^79^ Why NLRP9b does not interact with ASC,^79^ a finding which has also been observed in another overexpression study,^57^ may be multifactorial. Notably, mouse NLRP9b has less than 50% sequence similarity to human NLRP9.^77^ Further, inefficient expression of NLRP9b or the lack of co‐activating factors in HEK293T cells or their derivatives may be additional possibilities. Indeed, human and mouse intestinal cells are enriched in the expression of RNA sensors, including NLRP6, which are known to control Rotavirus infection.^80^, ^81^ Whether NLRP9b functions synergistically with additional innate immune sensors to drive immune responses to Rotavirus infection requires further investigation.^77^, ^81^

Crystal structure studies further complicated the interpretation of NLRP9 as inflammasome‐forming. These studies showed that the PYD of human NLRP9 exists as a monomer and does not oligomerize with one another.^82^, ^83^ Further, the full‐length human NLRP9 overexpressed in HEK293T cells,^68^, ^82^ and chemical‐induced dimerization of the human NLRP9 PYD in HEK293T/17 cells in the presence of ASC and caspase‐1, do not drive the proteolytic cleavage of GSDMD or pro‐IL‐1β.^57^ These data perhaps suggest that NLRP9 can induce inflammasome assembly only in the presence of dsRNA or Rotavirus infection. The lack of self‐polymerization capability of the PYD of human NLRP9 could also be due to charge inversions causing repulsion between the PYD and a bent N‐terminal loop oriented towards the interior of the helical bundle of PYD, both of which exhibit autoinhibitory functions, preventing self‐oligomerization, filament formation, and subsequent NLRP9 inflammasome assembly.^82^, ^83^ Furthermore, the crystal and cryogenic electron microscopy structures of an ADP‐bound inactivated form of human NLRP9 lacking the PYD showed a closed NACHT,^84^ as seen with other NLRs.^85^ Approximately 10 residues on the C‐terminal side of human NLRP9 are folded back from the tip of the LRR region, which is speculated to drive interactions with other proteins and oligomer formation.^84^ Together, the findings of these structural studies provide insights into the activation mechanisms of human NLRP9.

Emerging evidence suggests that variation/s in the gene encoding NLRP9 is associated with inflammatory and infectious diseases, including juvenile idiopathic arthritis, *Helicobacter pylori* infection, urothelial carcinoma, Alzheimer's disease, multiple sclerosis, and colon cancer.^86^, ^87^, ^88^, ^89^, ^90^, ^91^ Whether NLRP9 directly contributes to the initiation, development, and/or progression of these diseases requires further investigations.

## 5 NLRP10

Human and mouse NLRP10, also known as NALP10, NOD8, PAN5, or PYNOD, are expressed most abundantly in the skin but also in the colon, heart, kidneys, and skeletal muscle.^32^, ^92^, ^93^ NLRP10 contains a PYD and NACHT but lacks a LRR (Figures 1 and 2).^92^ Owing to this atypical structure compared to most other inflammasome‐activating NLR proteins, NLRP10 was considered to have inflammasome‐independent or inflammasome‐inhibitory functions.^78^, ^94^ Earlier studies using either HEK293T cells overexpressing human NLRP10 or peritoneal macrophages isolated from transgenic mice overexpressing mouse NLRP10 found that these cells had reduced caspase‐1‐mediated release of IL‐1β.^92^, ^93^ Furthermore, in primary rat glial cells treated with the peptide amyloid beta, NLRP10 inhibits the formation of the NLRP3 inflammasome and subsequent IL‐1β release.^95^ The inflammasome‐inhibitory roles of NLRP10 were not observed in bone‐marrow‐derived dendritic cells and peritoneal macrophages from mice lacking NLRP10 treated with the NLRP3 inflammasome activator LPS and ATP, with these cells secreting similar levels of IL‐1β compared with cells from WT mice.^96^

**FIGURE 7:**
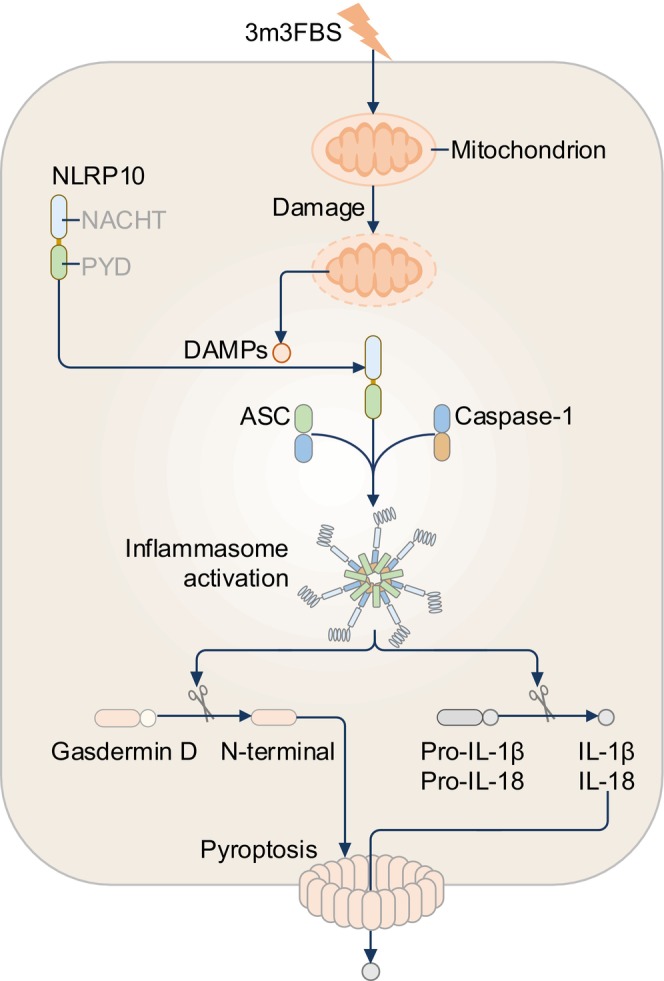
The NLRP10 inflammasome. NLRP10 senses mitochondria damage induced by the chemical 3 m3‐FBS and forms an inflammasome complex. In addition, the inflammation‐inducer dextran sodium sulfate, via an unknown mechanism, triggers the formation of an NLRP10 inflammasome in colonic epithelial cells of mice (not shown).

Compelling evidence has emerged that NLRP10 can assemble an endogenous inflammasome in human keratinocytes and the mouse intestine (Figure 7).^97^, ^98^ In response to the chemical 3 m3‐FBS that destabilizes mitochondria, NLRP10 induces ASC speck formation and caspase‐1‐mediated IL‐18 release in terminally differentiated human keratinocytes, HEK293T cells overexpressing NLRP10 and ASC, and mouse colonic organoids (Figure 7).^97^, ^98^ In these HEK293T cells, ASC speck formation and IL‐18 release increase in the presence of the TLR3 agonist poly(I:C).^97^ Although not a prerequisite for NLRP10 inflammasome formation, TLR3 activation sensitizes this process and implies the possibility that NLRP10 may also sense dsRNA. Given that the LRR, a putative ligand‐sensing domain for some NLR proteins, is absent in NLRP10,^97^, ^98^ the domain of NLRP10 that senses the ligand or perturbation would either be the NACHT or PYD. Further, the activation mechanism for NLRP10 may be similar to that of NLRP3 because the LRR of NLRP3 is not required for the activation of NLRP3.^99^ It is also possible that NLRP10 does not sense ligands directly and requires an adaptor molecule. In this case, NLRP10 may synergize with RNA sensors, such as RIG‐I, and/or RNA helicases DHX9 or DHX15 to form an inflammasome in the presence of dsRNA.

Unlike human keratinocytes, mouse macrophages treated with the chemical 3m3‐FBS do not assemble the NLRP10 inflammasome.^98^ In this context, damaged mitochondria may liberate mitochondrial DNA, a DAMP that triggers the formation of the AIM2 inflammasome in human and mouse macrophages.^100^ It is plausible that, in cell types that do not express AIM2, damaged mitochondria release DAMPs other than DNA that are sensed by NLRP10. These DAMPs may include mitochondrial reactive oxygen species (ROS), membrane proteins and/or lipids, or mitochondrial RNA.^101^ Identification of the ligands triggering the activation of NLRP10 would provide insights into the types of DAMPs that are released from damaged mitochondria and the molecular mechanisms of how NLRP10 respond to these DAMPs.

The physiological roles of the NLRP10 inflammasome have been explored in mouse models.^97^ Mice lacking NLRP10 only in intestinal epithelial cells are more susceptible to the inflammation‐inducer dextran sodium sulfate, and have reduced caspase‐1 activation and IL‐18 in the colon tissue compared to control mice,^97^ suggesting that the NLRP10 inflammasome from epithelial cells protects against intestinal damage. Whether genetic variants in NLRP10 predispose humans to inflammatory bowel disease is not known, but genetic variants of NLRP10 have been linked to an increased risk of developing the inflammatory skin disease atopic dermatitis.^102^, ^103^ Of these variants, a substitution of arginine 243 with tryptophan, an R243W variant, of NLRP10 hampers the ability of NLRP10 to assemble an inflammasome,^98^ hinting that NLRP10 might protect against skin inflammation. Given that loss‐of‐function in NLRP10 promotes skin inflammation in humans and that mice lacking NLRP10 develop intestinal inflammation,^97^, ^98^ strategies aimed at activating NLRP10 could lead to new anti‐inflammatory therapies.

Further studies revealed other opposing functions of NLRP10 in infectious and inflammatory diseases. For instance, in mice challenged with the fungus *Candida albicans*,^104^ the bacterium *Mycobacterium tuberculosis*,^105^ or the parasite *Leishmania major*,^106^ NLRP10 restricts the release of proinflammatory cytokines IL‐1β, IL‐6, and TNF. By contrast, in mice challenged with the bacterium *Shigella flexneri* or subjected to chemical‐induced skin contact hypersensitivity,^107^, ^108^ NLRP10 enhances the release of IL‐1β, IL‐6, and IL‐8 and promotes inflammation. It is possible that the expression of NLRP10 and/or the trigger‐specific activation of NLRP10 contributes to different biological outcomes.^109^ Further, the presence or absence and the magnitude of mitochondrial damage induced by these pathogens could be different and, in part, be underpinned by the use of different model systems and routes of infection.

## 6 NLRP11

**FIGURE 8:**
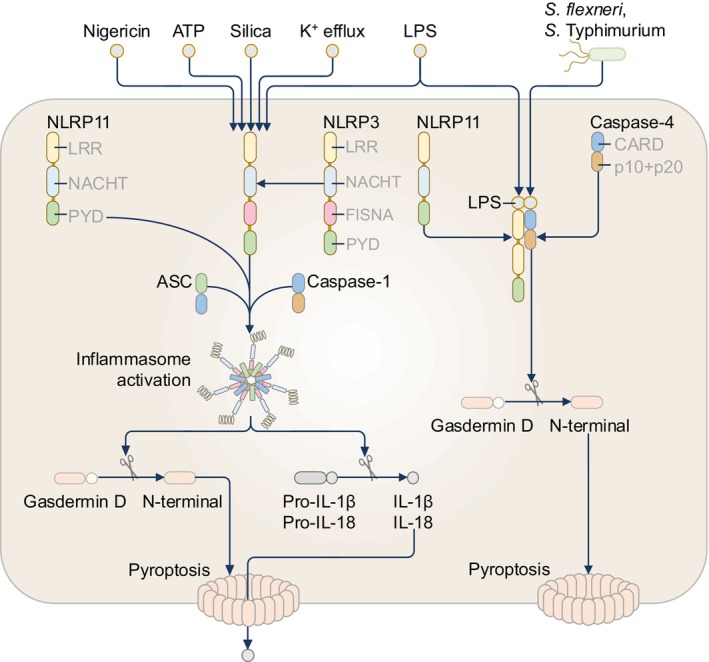
The NLRP11 inflammasome. NLRP11 forms a protein complex with NLRP3 and mediates NLRP3 inflammasome activation in human THP1 cells in response to the activators nigericin, adenosine triphosphate (ATP), silica, potassium ion (K^+^) efflux, and cytosolic lipopolysaccharide (LPS). Infection with the bacterium *Shigella flexneri* or *Salmonella enterica* serovar Typhimurium leads to the liberation of cytosolic LPS. LPS directly binds to both NLRP11 and caspase‐4, forming a complex. Caspase‐4 induces the proteolytic cleavage of gasdermin D that triggers pyroptosis.

NLRP11 (also known as NALP11, NOD17, PAN10, and PYPAF6) is an enigmatic NLR protein with its expression found in maturing oocytes from humans and rhesus macaque monkeys, and to a lesser extent in the monocytic cell line THP‐1, B cell lymphoma cell line Daudi, and primary CD19^+^ B cells derived from humans.^65^, ^110^, ^111^, ^112^, ^113^ Mice do not encode a gene for NLRP11. Emerging evidence suggests that NLRP11 contributes to inflammasome activation in human macrophages (Figure 8).^114^, ^115^ NLRP11 forms a complex with NLRP3 and ASC and mediates NLRP3 inflammasome activation in human THP1 cells in response to the NLRP3 activators nigericin, ATP, and silica, and also the caspase‐4 activator cytosolic LPS (Figure 8).^114^ The PYD of NLRP11 recruits ASC and induces ASC polymerization in synergy with NLRP3.^114^ Further, NLRP11 promotes the spontaneous oligomerization of constitutively‐active NLRP3 arising from a R260W mutation found in patients with cryopyrin‐associated periodic syndrome (CAPS).^114^

A later study has shown that, in human macrophages, NLRP11 is not required for nigericin‐induced NLRP3 activation, but instead, only mediates LPS‐induced caspase‐4 activation.^115^ Co‐immunoprecipitation of biotin‐conjugated LPS from the lysates of HEK293T cells overexpressing NLRP11 in the absence of caspase‐4 identified NLRP11 as an LPS‐binding partner, suggesting NLRP11 potentially functions as a PRR or co‐receptor of cytosolic LPS.^115^ The LPS‐sensing inflammasome complex is currently defined by human caspase‐4, human caspase‐5, or mouse caspase‐11 binding to cytosolic LPS.^7^, ^8^ NLRP11 also interacts with caspase‐4 and caspase‐1, but not with caspase‐5 and caspase‐9, in HEK293T cells.^115^ During infection with the bacterium *S*. *flexneri*, THP1 macrophages undergo caspase‐4 and gasdermin D activation in a NLRP11‐dependent manner,^115^ positioning NLRP11 as part of the non‐canonical inflammasome pathway (Figure 8). While there is no consensus on whether NLRP11 contributes to the activation of the canonical NLRP3 inflammasome pathway, these studies raised the question of why multiple receptors are required in mammalian cells to sense cytosolic LPS. During bacterial infection, LPS and other bacterial ligands from bacterial cells are liberated in the cytoplasm and recognized by interferon‐inducible guanylate‐binding proteins (GBPs).^116^ Human GBP1 has been shown to bind the LPS of *S*. *flexneri* in the cytoplasm of HeLa cells, facilitating the activation of caspase‐4.^117^ Given that *S*. *flexneri* can degrade human GBP1,^118^ it is possible that NLRP11 serves as another PRR that can recognize cytosolic LPS in response to pathogens that can inactivate GBPs. Another possibility is that NLRP11 mediates inflammasome activation when the cytoplasmic quantity of free LPS is low.^115^ Indeed, the amount of cytoplasmic LPS found in HeLa cells during *S*. Typhimurium infection is six times lower than that using electroporation.^8^

NLRP11 also interacts with other inflammasome‐associated components. Endogenous NLRP11 interacts with ASC in the human B cell lymphoma cell line Daudi, however, the functional outcome of this interaction is unknown given that this interaction does not affect caspase‐1 activation.^119^ In HeLa and HEK293T cell lines, the LRR of NLRP11 interacts with and sequesters the RNA helicase DDX3X, resulting in increased production of type I interferons and decreased NLRP3‐mediated caspase‐1 activation.^120^ How NLRP11 is able to execute potentially opposing functions is not known. The different configurations of NLRP11 signaling hubs in association with NLRP3, ASC, or DDX3X could potentially determine differential activation of immune signaling pathways. Indeed, domain specificity is central to ligand detection and autoregulation of certain NLRs in mounting an immune response. In humans, de novo duplication of the gene encoding NLRP11 is associated with systemic‐onset juvenile idiopathic arthritis.^86^ Further, NLRP11 interacts with and stabilizes the cytoskeleton protein vimentin, promoting proliferation and metastasis of human lung adenocarcinoma cells.^121^ Since mice do not carry a gene encoding NLRP11, studies elucidating the functional relevance of NLRP11 have been limited to cell culture studies in human cells. The generation of humanized mice expressing NLRP11 in a cell‐type‐specific manner could provide insights into the physiological functionality of NLRP11.

## 7 NLRP12

**FIGURE 9:**
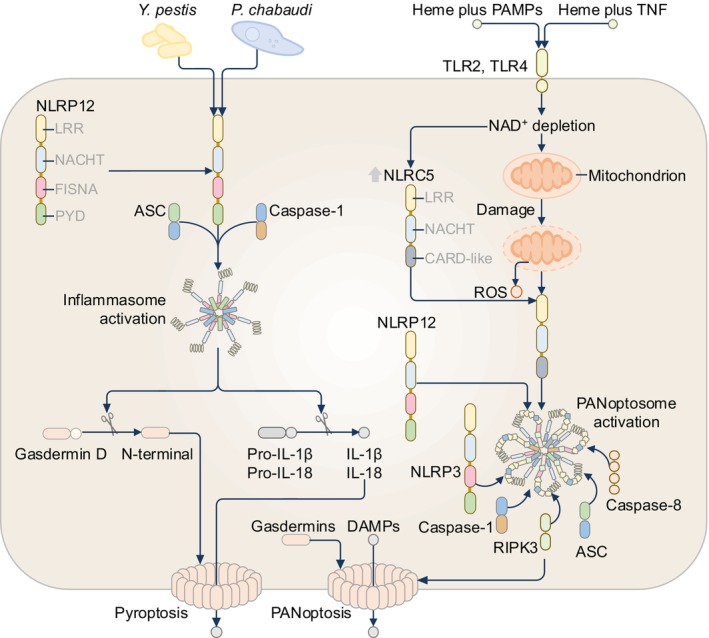
The NLRP12 inflammasome and PANoptosome. Infection with *Yersinia pestis* and *Plasmodium chabaudi* triggers the formation of the NLRP12 inflammasome in mouse macrophages. The combination of the iron‐containing molecular component of the hemoglobin protein and pathogen‐associated molecular patterns (PAMPs) or cytokines tumor necrosis factor (TNF) triggers the activation of Toll‐like receptors (TLR) 2 and TLR4, driving the depletion of cytoplasmic nicotinamide adenine dinucleotide (NAD^+^). The depletion of NAD^+^ upregulates the expression of the innate immune sensor NLRC5 (indicated by an upward arrow) and induces mitochondrial stress leading to the production of reactive oxygen species (ROS) and the formation of a PANoptosome complex comprising NLRP12, NLRC5, NLRP3, ASC, caspase‐1, caspase‐8, and the serine/threonine‐protein kinase RIPK3. The PANoptosome complex triggers the activation of the lytic inflammatory cell death pathway called PANoptosis. The lytic nature of PANoptosis is mediated by membrane‐disrupting proteins called gasdermins allowing damage‐associated molecular patterns (DAMPs) to be released from the dying cell.

NLRP12 (also known as NALP12, MONARCH‐1, PYPAF‐7, and RNO) is expressed in immune cells such as BMDMs, neutrophils, dendritic cells, and granulocytes of humans and mice.^122^, ^123^, ^124^, ^125^ NLRP12 is thought to be a negative regulator of inflammation, but evidence has emerged indicating a role for NLRP12 as an inflammasome or PANoptosome sensor in driving pro‐inflammatory responses (Figure 9).^124^, ^125^, ^126^ In response to *Yersinia* and *Plasmodium* infections in mouse macrophages, NLRP12 triggers caspase‐1 activation and the release of IL‐1β and IL‐18 (Figure 9).^127^, ^128^ The NLRP12 inflammasome is also activated in human corneal epithelial cells during herpes simplex virus (HSV)‐1 infection, triggering caspase‐1‐dependent IL‐1β and IL‐18 release.^129^ Furthermore, overexpression of NLRP12 can reduce HSV‐1 replication in the cornea owing to pyroptosis and IL‐18‐IFN‐γ‐mediated antiviral activity.^129^, ^130^ Overexpression and chemical‐induced dimerization of the PYD of human or mouse NLRP12 in HEK293T/17 cells in the presence of ASC and caspase‐1 leads to the proteolytic cleavage of gasdermin D and pro‐IL‐1β,^57^ confirming NLRP12 inflammasome activity. In contrast, another study showed that co‐expression of human NLRP12 and ASC in HEK293T cells does not lead to ASC speck formation,^68^ highlighting differences in experimental systems can yield drastically different phenotypic outcomes.

The microbial ligands activating NLRP12 are not known but given that NLRP12 appears to sense bacterial, protozoal, and viral pathogens, the ligands may be common amongst these microbes. A study analyzing cell death responses to PAMPs and DAMPs in mouse macrophages revealed that NLRP12 senses the combinations of LPS and heme, PAM3CSK4 and heme, or TNF and heme.^131^ NLRP12‐mediated sensing of these PAMPs and DAMPs leads to the formation of a PANoptosome complex comprising NLRP3, ASC, caspase‐1, caspase‐8, and RIPK3, driving innate immune lytic cell death and inflammation (Figure 9).^131^ In this case, both NLRP12 and NLRP3 are required to drive the proteolytic cleavage of caspase‐1 in response to LPS and heme.^131^ How the NLRP12 PANoptosome assembles remains unclear but NLRC5 has been identified as another NLR that contributes to PANoptosis in response to LPS and heme, PAM3CSK4 and heme, or TNF and heme.^132^ In this case, NLRP12 and NLRC5 are found within the same PANoptosome complex and can sense the depletion of cytosolic nicotinamide adenine dinucleotide (NAD^+^) in mouse macrophages (Figure 9).^132^ It is possible that an interaction between NLRP12 and NLRC5 provides a temporary protein scaffold that potentiates the recruitment of other PANoptosome components. Further, NLRC5 can sense NAD^+^ perturbation and ROS production induced by LPS and heme but how NLRP12 contribute to this process is still unclear.

NLRP12 can inhibit the NF‐κB pathway by directly interacting with the signaling protein IRAK1 or TRAF3, resulting in an attenuated release of proinflammatory cytokines and chemokines.^133^, ^134^, ^135^, ^136^ Further, NLRP12 can block both the canonical and non‐canonical NF‐κB pathways to avoid excessive production of inflammatory cytokines during the development of inflammatory bowel disease and colorectal cancer.^137^, ^138^, ^139^ In a hepatocellular carcinoma mouse model induced by the carcinogen diethylnitrosamine, NLRP12 has been shown to hinder the progression of hepatocellular carcinoma by reducing inflammation and limiting the proliferation of hepatocytes.^140^ The absence of NLRP12 leads to increased tumor burden, inflammation, and hepatocyte proliferation due to increased expression of pro‐proliferative genes, including *Myc*, *Ccnd1*, *MKi67*, and *Survivin*.^140^ These negative regulatory mechanisms of NLRP12 are detrimental to the host during certain infections, for example with *S*. Typhimurium exploiting NLRP12‐mediated inhibition of NF‐κB and ERK activation and anti‐microbial cytokine production,^141^ resulting in enhanced pathogen survival and persistence in the host tissue.^141^, ^142^, ^143^ Studies into SARS‐CoV‐2 also revealed a link between NLRP12 and virus infection. Two SARS‐CoV‐2‐genome‐encoded proteins, 3‐chymotrypsin‐like protease (3CLpro) and non‐structural protein 5 (NSP5), can cleave NLRP12, hampering the ability of NLRP12 to negatively regulate inflammation responses.^144^ This process has been linked to hyperinflammation in patients with severe COVID‐19.^144^ Across infection, inflammatory disease, and cancer, there is accumulating evidence indicating NLRP12 as an inflammasome and PANoptosome sensor, or as an inhibitory sensor of inflammation.

## 8 CARD8

Caspase recruitment domain‐containing protein 8 (CARD8) is also known as CARD‐inhibitor of NF‐kappa‐B‐activating ligand (CARDINAL) or tumor‐up‐regulated CARD‐containing antagonist of caspase 9 (TUCAN).^145^, ^146^ The gene encoding CARD8 is found in humans but not in rodents and was first reported as an antiapoptotic protein.^145^ CARD8 and NLRP1 share a similar C‐terminal domain organization, including a CARD, and a Function‐to‐Find domain (FIIND) consisting of ZU5 and UPA domains (Figure 1). The domain similarity between CARD8 and NLRP1 provided an early indication that CARD8 could also be an inflammasome sensor (Figure 1).

CARD8 can be activated by chemical and biological agents. In response to inhibition of the dipeptidyl peptidase DPP8 or DPP9, CARD8, and caspase‐1 were found to interact and assemble an inflammasome complex to induce pyroptosis.^147^ Chemical inhibitors of DPP8 or DPP9, such as the nonselective inhibitor of serine proteases Val‐boroPro (VbP), trigger CARD8 activation in naïve and resting T cells.^148^, ^149^ In addition to chemical inhibitors, the enzymatic activity of viral proteases can also trigger CARD8 activation.^150^ For example, HIV‐1 proteases can activate the CARD8 inflammasome in CD4^+^ T cells, driving the deletion of the CD4^+^ T cell population.^150^, ^151^ Similarly, the Coxsackie virus B3 2Apro and 3Cpro proteases and the coronavirus 3CL protease have also been reported to activate CARD8 in endothelial cells.^152^, ^153^ Additional CARD8 activators including reductive stress and protein folding stress have been identified.^154^, ^155^

**FIGURE 10:**
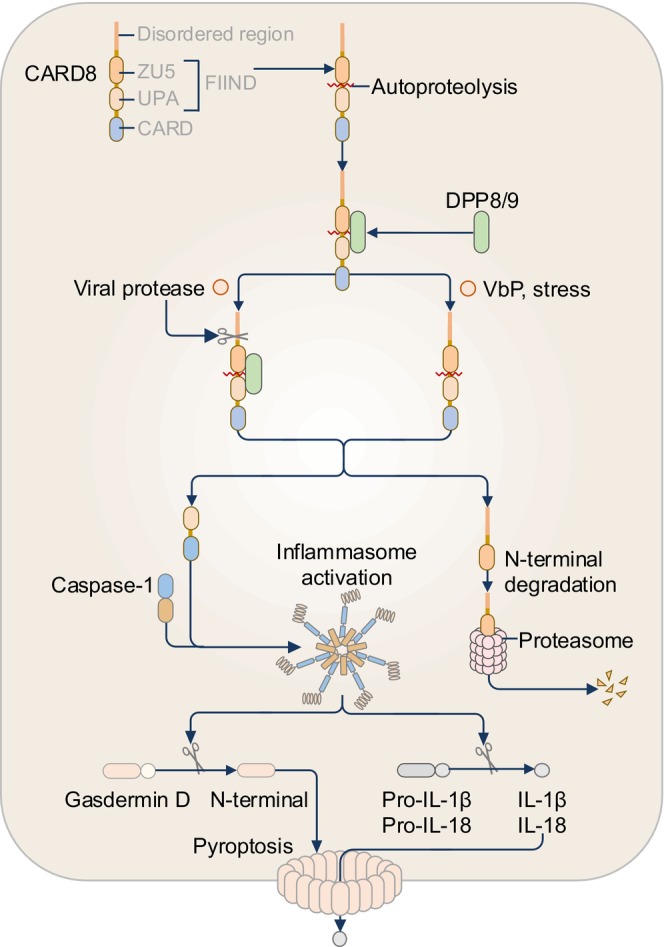
The CARD8 inflammasome. Under homeostatic conditions, CARD8 undergoes autoproteolysis and is restrained from activation following binding to the dipeptidyl peptidase DPP8 or DPP9. The enzymatic activity of viral proteases from HIV‐1 and Coxsackie virus triggers the proteolytic cleavage of the disordered region of CARD8, leading to the liberation of the C‐terminal fragment which recruits caspase‐1 and assembles the CARD8 inflammasome complex. The chemical Val‐boroPro (VbP) or stress releases DPP8 or DPP9, exposing the N‐terminal fragment for proteasomal degradation and liberating the C‐terminal fragment for inflammasome assembly.

Under homeostatic conditions, CARD8 undergoes an autoproteolysis event within its FIIND at position 296, resulting in two fragments: an N‐terminal fragment containing a 160‐amino‐acid disordered region and the FIIND ZU5 domain, and a C‐terminal fragment comprising the FIIND UPA domain and CARD (Figure 10).^156^ These fragments remain non‐covalently attached and also interact with DPP8 or DPP9 which restrains inflammasome formation.^157^, ^158^, ^159^ CARD8 activators disrupt this equilibrium, with viral proteases cleaving the disordered region within the N‐terminus or sequestration of DPP8 or DPP9 by VbP.^147^, ^150^ These CARD8 activators expose the N‐terminal fragment for 20S proteasomal degradation, fully liberating the C‐terminal fragment.^160^, ^161^ Once liberated, C‐terminal fragments form filaments, and via their CARD, recruit caspase‐1 and assemble an inflammasome complex (Figure 10).^162^, ^163^, ^164^

CARD8 can also serve as an immune checkpoint by negatively regulating other innate immune sensors. CARD8 can directly interact with and suppress the activation of NLRP1 and NLRP3 inflammasomes, and inhibit NOD2 signaling, likely controlling aberrant activation.^165^, ^166^, ^167^, ^168^ CARD8 has multiple isoforms, with the T‐48 isoform of CARD8 being identified first. Other isoforms called T‐47, T‐51, T‐54, and T‐60 have been reported. These isoforms vary amongst their N‐terminal lengths, resulting in differing molecular sizes,^145^, ^146^, ^167^, ^169^ and may potentially explain how CARD8 is able to respond to different types of PAMPs and DAMPs, and also carry out inhibitory functions. The expression pattern and the functional properties of these isoforms across different cell types remain to be characterized. Mutations in the gene encoding CARD8 have been implicated in the susceptibility of inflammatory diseases such as atherosclerosis, rheumatoid arthritis, tuberculosis, and inflammatory bowel disease,^170^, ^171^, ^172^, ^173^, ^174^ though many of these associations require further experimental validation. Further studies identifying novel inhibitors of CARD8 may help prevent aberrant CARD8 activation in inflammatory diseases.

## 9 MxA

**FIGURE 11:**
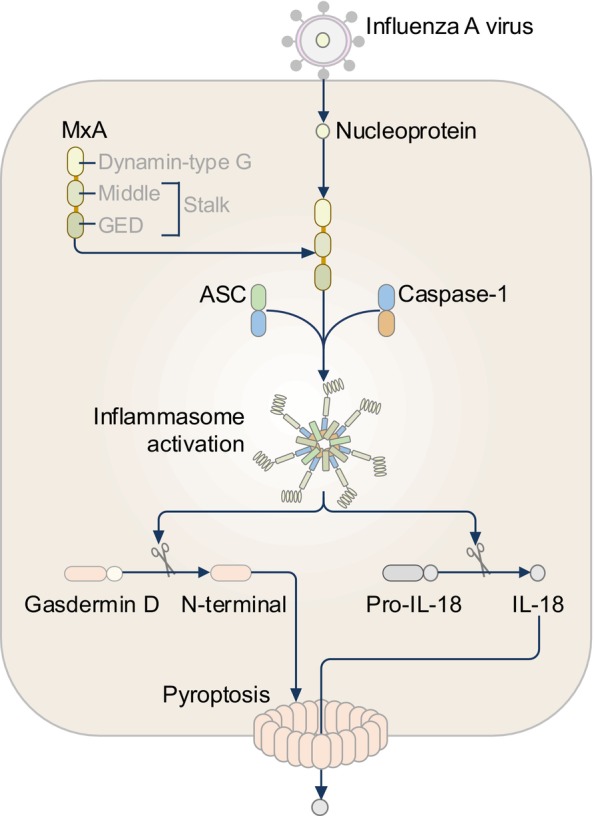
The MxA inflammasome. The influenza A virus (IAV)‐derived nucleoproteins are recognized by human Myxovirus resistance protein 1 (also called MxA). The GTPase domain (also called the Dynamin‐type G domain) of MxA interacts with the adaptor protein apoptosis‐associated speck‐like protein containing a caspase‐recruitment domain (also known as ASC or PYCARD), leading to the oligomerization of ASC, resulting in the formation of the MxA inflammasome.

Myxovirus resistance protein 1 (MX1, also known as MxA and IFI‐78K) belongs to the dynamin superfamily of large GTPases and is evolutionarily conserved amongst most vertebrates.^175^, ^176^, ^177^ The expression of MxA is induced by type I and type III interferons.^178^ MxA is an antiviral protein in mammalian cells with roles against pathogenic influenza A virus (IAV).^178^, ^179^ While the antiviral property of MxA has long been recognized,^178^, ^179^ the link between MxA and inflammasome signaling was only identified in 2019.^180^ A high‐content short hairpin RNA library screen identified that human MxA forms an inflammasome in immortalized human respiratory epithelial cell line PL16T following infection with IAV (Figure 11).^180^ Immunoprecipitation of ASC revealed that the GTPase domain (also known as the dynamin‐type G domain), but not the Stalk, of MxA interacts with the PYD of ASC in PL16T cells infected with IAV.^180^ The binding between the GTPase domain of MxA and ASC triggers MxA oligomerization and the subsequent recruitment and oligomerization of ASC, resulting in the formation of the MxA inflammasome.^180^ Moreover, MxA induces the formation of ASC specks in response to IAV nucleoproteins, but not to IAV polymerases,^180^ pointing out that MxA might sense viral nucleoproteins (Figure 11).

Similar to human MxA, porcine Mx1 assembles an inflammasome in PL16T cells infected with IAV, whereas murine Mx1 does not.^180^ A potential incompatibility between mouse proteins and a human cell line may provide a possible explanation. Further, mouse Mx1 is expressed in the nucleus of cells and inhibits the growth of viruses that replicate in the nucleus, such as tick‐transmitted Thogoto virus and H5N1 influenza viruses,^181^, ^182^, ^183^ whereas human MxA is predominantly expressed in the cytoplasm and restricts the growth of a broad range of viruses including IAV, vesicular stomatitis viruses, and La Crosse virus.^180^, ^184^, ^185^, ^186^ The differential subcellular locations between mouse Mx1 and human MxA might also in part explain why human MxA but not mouse Mx1 is able to form an inflammasome.

In transgenic mice expressing human MxA and infected with IAV, MxA colocalizes with ASC specks in bronchiolar epithelial cells,^180^ providing evidence that MxA assembles a physiological inflammasome. In human THP1 macrophages infected with IAV, however, the release of IL‐1β requires NLRP3 rather than MxA,^180^ arguing that MxA does not function in certain immune cells. Since the innate immune sensor ZBP1 (also known as DAI) can also sense IAV nucleoproteins in non‐immune cells,^187^ it would be intriguing to investigate whether ZBP1 and MxA are co‐receptors and can potentially form a MxA‐ZBP1 inflammasome complex. Investigating whether other PAMPs and DAMPs can activate the MxA inflammasome would indicate a broader role of MxA in innate immunity.

## 10 CONCLUDING REMARKS AND FUTURE PROSPECTS

The field of inflammasome biology is rapidly expanding with some inflammasome sensors being well established, while many others have just begun to be explored. Given the structural homology of NLR proteins, it is possible that additional NLR proteins are inflammasome‐forming sensors, which are to be identified in specific cell types in response to specific activators. Differences in findings between overexpression systems and endogenous cellular and mouse models have made it difficult to interpret whether a specific inflammasome can genuinely form. Further, some NLR proteins, such as NLRP12 or NLRC5, can form larger PANoptosome protein complexes that resemble the scaffold of inflammasome complexes. Discoveries of these higher‐order multi‐sensor structures may indicate that prior studies reporting putative inflammasome functions for specific PRRs may merely be an incomplete snapshot of the PANoptosome response. Future investigations into these emerging inflammasome sensors, their ligands, and insights into their structures and activation mechanisms could provide a broader and holistic perspective regarding their importance in health and disease. Moreover, the identification of genetic variations in these emerging inflammasome sensors and their associations with human diseases could provide biological clues into their functions. Currently, no inhibitors are available that can target a specific inflammasome sensor with high‐level specificity and potency, other than those for NLRP3. Drug screening programs would in part address this gap in linking fundamental inflammasome biology to therapeutic applications.

## FUNDING INFORMATION

Work from our laboratory is supported by the National Health and Medical Research Council of Australia under Ideas Grant APP2002686 and Investigator Grant number 2026910, The Australian Government Australia‐India Strategic Research Fund Collaborative Research Project AIRXV000005, and a CSL Centenary Fellowship to SMM.

## CONFLICT OF INTEREST STATEMENT

The authors declare no conflict of interest.

## Data Availability

Data sharing not applicable to this article as no datasets were generated or analyzed during the current study.
